# Proteomic Analysis Identifies FNDC1, A1BG, and Antigen Processing Proteins Associated with Tumor Heterogeneity and Malignancy in a Canine Model of Breast Cancer

**DOI:** 10.3390/cancers13235901

**Published:** 2021-11-24

**Authors:** Yonara G. Cordeiro, Leandra M. Mulder, René J. M. van Zeijl, Lindsay B. Paskoski, Peter van Veelen, Arnoud de Ru, Ricardo F. Strefezzi, Bram Heijs, Heidge Fukumasu

**Affiliations:** 1 Laboratory of Comparative and Translational Oncology, Department of Veterinary Medicine, School of Animal Science and Food Engineering, University of São Paulo, Pirassununga 13635-900, Brazil; yo.gcordeiro@gmail.com (Y.G.C.); lindsaybiom@usp.br (L.B.P.); rstrefezzi@usp.br (R.F.S.); 2 Center of Proteomics and Metabolomics, Leiden University Medical Center, 2300 RC Leiden, The Netherlands; leandra.mulder@live.nl (L.M.M.); R.J.M.van_Zeijl@lumc.nl (R.J.M.v.Z.); P.A.van_Veelen@lumc.nl (P.v.V.); A.H.de_Ru@lumc.nl (A.d.R.); B.P.A.M.Heijs@lumc.nl (B.H.)

**Keywords:** breast cancer, canine model, comparative oncology, MALDI imaging, tumor progression

## Abstract

**Simple Summary:**

Comparative oncology is centered around the study of naturally occurring tumors in animals as a parallel and complementary model for human cancer research. Canine mammary tumors pose as excellent models since they share similarities in their spontaneous nature, histological subtypes, genetic background, and clinical course, which would be impossible to reproduce in murine models. Our study aimed to investigate cancer heterogeneity in primary tumors and metastasis, by applying bottom-up proteomics and mass spectrometry imaging to identify potential disease-state markers. We have demonstrated that the malignant phenotype may have arisen as a consequence of alterations in the expression of proteins involved in immune evasion facilitating metastatic events. To our knowledge, this is the first study to use mass spectrometry imaging in a dog model of breast cancer, that have demonstrated that poorly described proteins might play important roles in cancer spreading and should be further validated as potential early-stage tumor biomarkers.

**Abstract:**

New insights into the underlying biological processes of breast cancer are needed for the development of improved markers and treatments. The complex nature of mammary cancer in dogs makes it a great model to study cancer biology since they present a high degree of tumor heterogeneity. In search of disease-state biomarkers candidates, we applied proteomic mass spectrometry imaging in order to simultaneously detect histopathological and molecular alterations whilst preserving morphological integrity, comparing peptide expression between intratumor populations in distinct levels of differentiation. Peptides assigned to FNDC1, A1BG, and double-matching keratins 18 and 19 presented a higher intensity in poorly differentiated regions. In contrast, we observed a lower intensity of peptides matching calnexin, PDIA3, and HSPA5 in poorly differentiated cells, which enriched for protein folding in the endoplasmic reticulum and antigen processing, assembly, and loading of class I MHC. Over-representation of collagen metabolism, coagulation cascade, extracellular matrix components, cadherin-binding and cell adhesion pathways also distinguished cell populations. Finally, an independent validation showed FNDC1, A1BG, PDIA3, HSPA5, and calnexin as significant prognostic markers for human breast cancer patients. Thus, through a spatially correlated characterization of spontaneous carcinomas, we described key proteins which can be further validated as potential prognostic biomarkers.

## 1. Introduction

The estimated number of human breast cancer cases is still increasing worldwide, accounting for 11.6% of all new cases of cancer and 6.6% of deaths due to the disease until 2018 [1,2]. Even with a thorough histological examination and the use of well-recognized gene expression patterns for molecular subtyping [3], a reliable classification for clinical prognosis and therapy decision making might be challenging. In order to find new prognostic markers and develop more efficient and precise treatments, a deeper molecular understanding of the disease is necessary.

To gain new insights into tumor growth and revolutionary therapy research, a trustworthy animal model that mimics the fundamental aspects of human cancer is required. In this context, spontaneous canine mammary tumors are an excellent example of the intricate biology behind heterogeneous cancer progression. Pet dogs not only experience the same environment and risk factors as people, but they also share anatomical and histological similarities, comparable clinical features, as well as more homologous DNA and protein sequences compared to rodent models [4,5,6,7]. Moreover, the large population of dogs and improvements in the pet–owner relationship can simultaneously benefit both sides by helping researchers to improve the assessment of novel treatments for humans by treating pet animals with cancer [5].

It has been recognized that cancer is hardly a disease of tumor cells solely, but instead a disorder of imbalance in which tumor microenvironment plays crucial roles in development and malignancy [8,9]. Mass spectrometry imaging (MSI) enables the spatially correlated, label-free, and highly multiplexed molecular analysis directly from histological sections, and therefore is a useful tool for analyzing tumor heterogeneity in cancer tissues [10]. One advantage of MSI is the possibility of investigating cancer cells simultaneously with their immediately adjacent microenvironment, reducing the interference of other extra-tumor stroma components which could lead to misinterpretations. Despite limitations, such as the restricted analytical depth and the challenging high-dimensional datasets [11], the combination of bottom-up and top-down proteomics approaches, achieved by combining MSI and LC-MS/MS, represents a valuable tool for basic and translational oncology research [12].

Therefore, we aimed to explore tumor heterogeneity and molecular alterations in pathways involved in cancer progression, which could favor diagnosis, prognosis, and therapeutic strategies. To date, only a few studies have employed MSI in canine cancer, all of which comprised lipid profiling to identify molecules that might be used to discriminate between tumor types, or tumoral and non-tumoral areas [13,14,15]. To our knowledge, this is the first study to explore tumor heterogeneity using MSI in a dog model of breast cancer. Here, we have identified variations in peptide expression between cell populations in different degrees of differentiation, therefore malignancy, which may lead to the discovery and improvement of potential biomarkers.

## 2. Materials and Methods

### 2.1. Tissue Samples

All procedures were approved by FZEA/USP Ethics Committee (CEUA nº 8632101017). Mammary tumor samples from five unspayed dogs submitted to elective mastectomies were obtained in a collaboration with the Veterinary Hospital of the Faculty of Animal Science and Food Engineering (FZEA/USP) in Pirassununga, Brazil, and the Veterinary Hospital Dr. Vicente Borelli, from the Octávio Bastos Teaching Foundation (UNIFEOB) in São João da Boa Vista, Brazil. Patients had no previous history of either surgical or chemotherapy treatments.

### 2.2. TMA Construction

All tumor samples were divided into smaller fragments of ≅ 1 cm^3^ after surgical excision. Fragments were fixed in a 10% buffered formalin solution and processed according to standard procedures. The diagnosis of mammary cancer was confirmed by histological evaluation following the classification proposed by Goldschmidt et al. [16].

For tissue microarray (TMA) arrangement, formalin-fixed, paraffin-embedded (FFPE) blocks were sectioned into 4 μm thick pieces, hematoxylin and eosin (HE) stained, and regions were marked for a good depiction of intratumor heterogeneity across the solid mass. Areas containing epithelial and myoepithelial cells, as well as stromal regions including vessels, inflammatory cells, and connective tissues were chosen. From each tumor, up to 5 punches/cm^3^ were taken, and the 2 mm cores were assembled with at least 1 mm spacing between samples. The number of cores varied between 40 and 56 cores per TMA. In addition, metastatic cells in lymph nodes or other secondary sites (i.e., lungs) as well as non-neoplastic mammary glands from different animals were also placed asymmetrically at one end of each block for control purposes. After assembly, TMAs were sectioned and stained with HE to assess the integrity of the samples.

### 2.3. Mass Spectrometry Imaging

For matrix-assisted laser desorption/ionization mass spectrometry imaging (MALDI-MSI), FFPE TMAs were sectioned into 4 μm-thick sections and mounted on indium-tin-oxide (ITO)-coated glass slides (Bruker Daltonics, Bremen, Germany) additionally coated with 0.05% poly-L-lysine (Sigma-Aldrich, St. Louis, MO, USA) and 0.1% Nonidet P-40 (Sigma-Aldrich, St. Louis, MO, USA). Sections were dewaxed in xylene, rehydrated, and washed with deionized water after an hour on a hot plate at 60 °C. Heat-induced antigen retrieval was performed in citrate buffer (10 mM at pH 6) at 121 °C for 20 min, then cooled to room temperature for 120 min. Trypsin (0.02 μg/mL in deionized water, Trypsin Gold, Promega Corporation, Wisconsin, WI, USA) was applied for tissue digestion using the SunCollect (SunChrom, Friedrichsdorf, Germany) automated pneumatic spray platform (15 layers at 10 μL/min). Slides were then incubated for 18 h using the SunDigest (SunChrom) incubator at 37 °C and 95% of relative humidity. Following digestion, MALDI-matrix (25 mg/mL of 2,5-dihydroxybenzoic acid in 50% aqueous acetonitrile and 0.1% trifluoroacetic acid) was also applied using the SunCollect (7 layers at (1) 10 μL/min, (2) 20 μL/min, (3) 30 μL/min, (4+) 40 μL/min).

MALDI-FT-ICR-MSI data acquisition was performed in positive ion mode on a 9.4 T solariX equipped with CombiSource™ and dynamically harmonized ParaCell™ (Bruker Daltonics). Data were acquired over an *m/z* range between 600–3500 Da, 200 laser shots per pixel, and the small laser focus (~50 μm diameter) with a pixel size of 100 × 100 μm^2^. Instrument calibration was performed using Peptide Calibration Standard (PepMix, Bruker Daltonics).

### 2.4. Histopathological Staining and Annotation

Excess MALDI-matrix was removed with 70% ethanol after MALDI-MSI. TMA slides were stained with HE and later scanned (Philips Slide Scanner, Andover, MA, USA) on 5, 10, 20, and 40× magnification. Cores that were eventually lost or damaged during tissue processing and/or staining were not included in the analyses. Using flexImaging (v5.0, Bruker Daltonics), scanned images were co-registered to the MSI data allowing for virtual microdissection. The annotation of regions of interest (ROIs), mainly comprised cancer epithelial cell populations. Annotation step avoided major vessels and ducts along with necrotic and desmoplastic areas, in order to minimize the interference caused by the influence of MALDI-matrix signals accumulating in empty spaces. Tumor ROIs were categorized into two different groups based on morphological criteria of the malignancy of canine mammary cancer adapted from the well-established grading system published by Goldschmidt et al. [16], as follows: (i) well-differentiated tumor populations (WD), presenting uniform cell morphology to a moderate degree of cell and nuclei pleomorphism, none to occasional hyperchromatic nuclei and none to occasional nucleoli. Differentiated tubules in >25% of region area and rare to occasional mitoses. (ii) poorly differentiated tumor populations (PD), showing a high degree of pleomorphism, hyperchromatic nuclei, presence of prominent and/or multiple nucleoli. Differentiated tubules in <25% of the region assuming areas of solid growth, where frequent mitoses could be observed.

### 2.5. MALDI-MSI Data Processing

Raw data were exported as an imzML file for preprocessing and peak picking using the SCiLS Lab Software (v2016b, Bruker Daltonics, Bremen, Germany). In the R environment (www.r-project.org accessed 15 July 2021), data processing was conducted using the rMSIproc package [17]. Spectra were aligned using a maximum shift of 50 ppm, alignment iterations = 3, and alignment oversampling = 2. Data were root-mean-squared (RMS)-normalized, and peak picking was conducted setting a signal to noise ratio (S/N) = 7 and a binning tolerance of 12 scans. Peaks detected in less than 5% of the total number of spectra were removed. After statistical analysis (described in the following paragraph), the list with significant peaks was checked for the presence of isotope peaks using mMass 5.5.0 [18]. Deisotoping was performed using a max charge of 1+ and isotope mass tolerance of 0.02 *m/z* for monoisotopic mass selection.

### 2.6. Liquid Chromatography-Tandem Mass Spectrometry (LC-MS/MS)

For LC-MS/MS, tissue tryptic digestion was performed as previously described in the MALDI-MSI methods. A solution of 50% aqueous acetonitrile and 0.1% trifluoroacetic acid was used for peptides extraction from the TMAs. Then, extracted peptides were desalted using C18 ZipTips (Millipore, Billerica, MA, USA) according to the standard protocol, and later dried. Lyophilized peptides were dissolved in 95/3/0.1 (% *v/v/v*) water/acetonitrile/formic acid and analyzed by online C18 nano HPLC MS/MS with a system consisting of an Easy-nLC 1200 gradient HPLC system (Thermo Scientific, Bremen, Germany), and an Orbitrap Fusion Lumos mass spectrometer (Thermo Scientific). Next, the sample was injected onto a precolumn (100 μm × 15 mm, C18 Reprosil-Pur C18-AQ, 3 µm, 120 A, and eluted via a homemade analytical nano-HPLC column (50 cm × 75 μm; Reprosil-Pur C18-AQ 1.9 µm, 120 A (Dr. Maisch, Ammerbuch, Germany). The gradient was run from 2 to 36% solvent B (20/80/0.1 water/acetonitrile/formic acid (% *v/v/v*) in 120 min. The nano-HPLC column was drawn to a tip of ~10 μm acting as the electrospray needle of the MS source. The mass spectrometer was operated for a cycle time of 3 s in data-dependent MS/MS mode, with an HCD collision energy at 32 V and recording of the MS2 spectrum in the orbitrap, with a quadrupole isolation width of 1.2 Da. The resolution was set to 120,000, the scan range 400–1500, at an AGC target of 4,000,000 at a maximum fill time of 50 ms in the master scan (MS1). Precursors were dynamically excluded after *n* = 1 with an exclusion duration of 60 s, and with a precursor range of 20 ppm. The charge states 2–4 were included. For MS2, the first mass was set to 110 Da, and the MS2 scan resolution was 30,000 at an AGC target of 40,000 at a maximum fill time of 60 ms.

As post-analysis, raw data were converted to peak lists using Proteome Discoverer version 2.2 (Thermo Electron), and protein identification was carried out by comparing the lists to the Canis2019 database (24,669 entries) on Mascot v. 2.2.04 (www.matrixscience.com, accessed on 15 July 2021). Mascot searches were performed using 10 ppm and 0.02 Da tolerance for precursor and fragment mass, respectively, and trypsin was defined as the protease. Methionine oxidation and the main formaldehyde reaction product on Lysine (+12.000 *m/z*) were set as a variable modification, while Carbamidomethyl (C) was a fixed modification. Peptides with an FDR ≤ 1% in combination with a mascot ion score ≥25 were accepted. For peptide identity assignment (IDs), observed MALDI-MSI signals were matched to LC-MS/MS data considering a maximum mass error of ±10 ppm. Single peptides assigned to different proteins from the same family were kept since they may be helpful for a full comprehension of the results.

### 2.7. Statistical Analysis

#### 2.7.1. Discriminative *m/z* Signals

Groups were pre-adjusted so that the number of spectra obtained from each animal was randomly—and equally—sampled before statistical analysis. Discriminative peaks between annotated WD and PD tumor regions were examined using a receiver operating characteristic (ROC) analysis and then tested for significance using the Mann–Whitney Wilcoxon Test followed by Benjamini–Hochberg (BH) post-test correction. For that, the mean intensity of each *m/z* feature in every ROI was calculated and tested. Then, the same number of spectra from each condition (*n* = 3000) were used for the ROC curve, and signals with AUC ≥ 0.70 in at least three out of five ROC analyses were tested. Only *m/z* signals presenting a *p*-value ≤ 0.05 and FDR ≤ 0.05 were considered significant.

#### 2.7.2. Functional Enrichment Analysis

For a more comprehensive knowledge base about protein function, the human genome-coding database was employed as a reference set. Thus, gene ontology (GO) and pathway enrichment analysis were determined through the overrepresentation test on the PANTHER classification system (www.pantherdb.org, accessed 15 November 2021), using the REACTOME (version 65) database [19]. Protein symbols retrieved from peptides with unique matches were used as input and statistical analysis was performed using Fisher’s exact test followed by FDR correction. Enriched GO terms and pathways were considered significant if FDR ≤ 0.05.

#### 2.7.3. Independent Comparative Validation

To further evaluate the significance of relevant proteins found in canine samples as prognostic factors in human breast cancer, an in silico validation was performed using an online survival analysis tool [20] (www.kmplot.com, accessed 15 July 2021). The datasets include gene expression (Affymetrix microarrays) and survival data from Gene Expression Omnibus (GEO and The Cancer Genome Atlas (TCGA). Overall survival (OS) and distant metastasis-free survival (DMFS) were analyzed using protein IDs as input, and the optimal probe [21] was selected for the assessment of the expression levels. All possible cutoff values between the lower and upper quartile were computed, and the best performing threshold was used to divide patients into two different cohorts. No restrictions were made regarding tumor subtypes such as ER, PR, and HER2 status, grade, or stage. No restrictions were made regarding patient treatment. Survival curve, number-at-risk, hazard ratio (and 95% confidence intervals), and log-rank *p* were displayed on the Kaplan–Meier plot. A protein was considered a significant marker when *p*-value ≤ 0.05.

## 3. Results

### 3.1. Patients, Tumor Samples and Tissue Annotation

**Figure 1:**
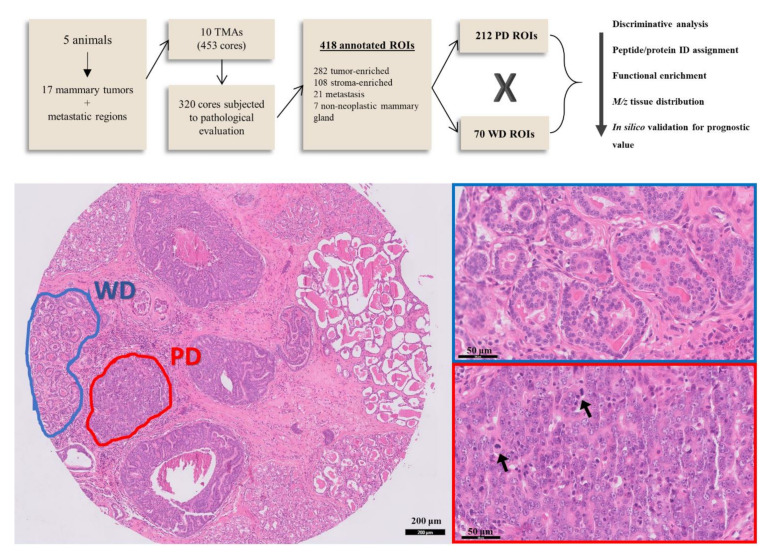
Peptide expression was compared between neoplastic cell populations in two distinct levels of differentiation (well-differentiated, WD, and poorly differentiated, PD), from 17 tumor samples assembled in FFPE tissue microarrays. Protein identity assignment of significant features was performed based on mass matching to an LC-MS/MS derived peptide database. Protein IDs were then submitted to functional enrichment analysis and promising prognostic marker candidates were validated in silico using a human gene expression database. HE stained TMA core shows the annotation of a WD (blue) and a PD (red) region of interest (ROI), detailed in the images on the right. ROIs were classified based on morphological criteria such as tubule differentiation, cell, nuclei, and nucleoli pleomorphism. Arrows point to atypical mitoses inside a PD tumor region.

Canine mammary gland FFPE tissue samples were examined using MSI. The study included 17 mammary tumors surgically removed from five female dogs diagnosed with metastatic disease, confirmed by positive anti-cytokeratin immunostaining in lymph nodes and other metastatic sites (patient information and histological classification of samples are detailed in Supplementary Table S1). Epithelial and myoepithelial populations, stromal areas consisting of connective tissue and peritumoral inflammatory cell infiltration, as well as metastatic regions and non-neoplastic glands were assembled in ten TMA blocks. A total of 453 cores, from which 320 were viable, were submitted to pathological classification and annotation. From annotated regions, a total of 282 tumor ROIs were selected and further categorized as WD and PD (Figure 1). We obtained 70 WD and 212 PD ROIs, along with 21 metastasis regions and seven ROIs of non-neoplastic mammary glands. In addition, 108 stroma-only regions were annotated, but not included in any statistical analysis.

### 3.2. Protein Identification and Discriminative m/z Signals between Low and High Grade Intratumor Regions

**Figure 2:**
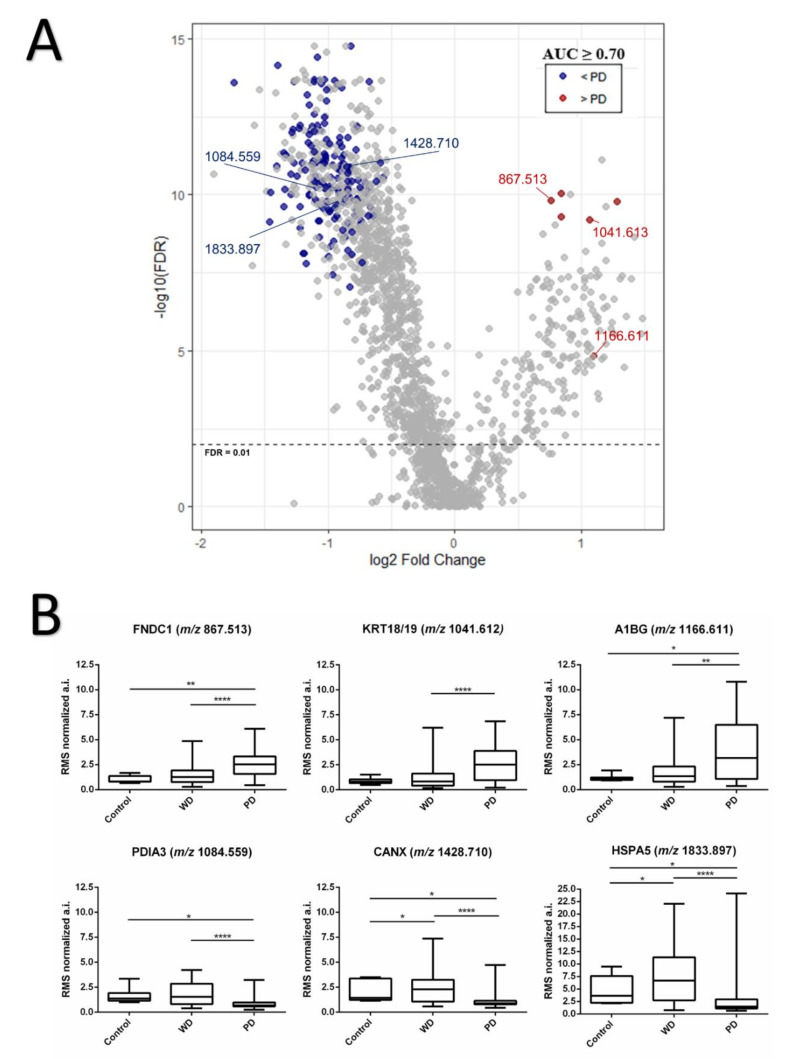
(**A**) Volcano plot shows the discriminative peaks (AUC ≥ 0.70 and FDR ≤ 0.05) in WD vs. PD group. Higher intensity levels in PD tumors are indicated in red, lower intensity levels are indicated in blue (**B**) Boxplot analysis of representative peptide expression of six discriminative *m/z* signals in canine mammary carcinoma tissues. Normalized absolute intensity data and error bars are shown for WD and PD tumor areas, as well as non-neoplastic mammary gland used as control. Ions at *m/z* 867.513, 1041.613, and 1166.611 were more expressed in PD ROIs, while signals at *m/z* 1084.559, 1428.710, and 1833.897 presented a lower expression in the same regions. (*) *p* < 0.05 (**) *p* < 0.01 (****) *p* < 0.0001.

After data processing and peak picking, a list containing 1780 *m/z* features was obtained from the examined tissues. Next, molecular dissimilarities between WD and PD subpopulations that could be contributing to tumor malignancy were determined. After deisotoping, 168 differentially expressed *m/z* signals were found (Supplementary Table S2), where the majority of features (162) were more intensely expressed in WD tumor regions compared to PD (Figure 2A).

Protein identity assignment of the 168 discriminating features based on mass matching to the LC-MS/MS derived peptide database resulted in 38 proteins IDs with unique matches (from 47 corresponding peptides), and 10 extra IDs from four signals which were assigned to two or more proteins belonging to the same family/type (keratin 18 and 19, KRT18/KRT19 at *m/z* 1041.613 with a mass error of 7.37 ppm; heat shock protein 90 alpha family class B member 1 and class A member 1, HSP90AB1/HSP90AA1 at *m/z* 1311.563 with a mass error of 5.11 ppm; tropomyosin 1 and 2, TPM1/TPM2 at *m/z* 1332.648 with a mass error of 7.42 ppm; and beta tubulins, TUBB4B/TUBB/TUBB2A/TUBB1 at *m/z* 1636.829 with a mass error of 0.42 ppm). A full list of identity assignments can be found in Supplementary Table S3. Peptides whose identities were assigned to Fibronectin type III domain containing 1 protein (FNDC1, at *m/z* 867.513, mass error = 9.61 ppm), Alpha-1B-glycoprotein (A1BG, at *m/z* 1166.611, mass error = 2.25 ppm), and double-matching keratins 18 and 19 (KRT18/KR19 at *m/z* 1041.613, mass error = 7.37 ppm) were among *m/z* signals showing higher intensity in PD regions (Figure 2B). Besides those peptides, ions at *m/z* 679.547, *m/z* 742.305, and *m/z* 1158.647 were found to be also overexpressed in PD regions, but the identities could not be retrieved from LC-MS/MS data.

### 3.3. Functional Enrichment Analysis of Differentially Expressed Peptides between Well and Poorly Differentiated Tumor Populations

To increase our understanding of biological and molecular pathways distinguishing WD and PD populations, gene ontology (GO) was performed using protein IDs retrieved from LC-MS/MS data of discriminative *m/z* signals between conditions. GO of unique IDs significantly enriched for terms such as protein folding in endoplasmic reticulum, extracellular matrix structural constituent, molecule activity, cadherin binding, and cell adhesion (Table 1).

Besides a general overview of GO processes, we also performed a functional pathway enrichment of differentially expressed peptides using the open-access and peer-reviewed REACTOME database, which describes possible reactions if all annotated proteins were present and active simultaneously in a cell. Pathways directly involved in collagen metabolism, coagulation cascade, extracellular matrix proteoglycans, and signaling by receptor tyrosine kinase showed as significant in enrichment analysis (Table 2).

**Figure 3:**
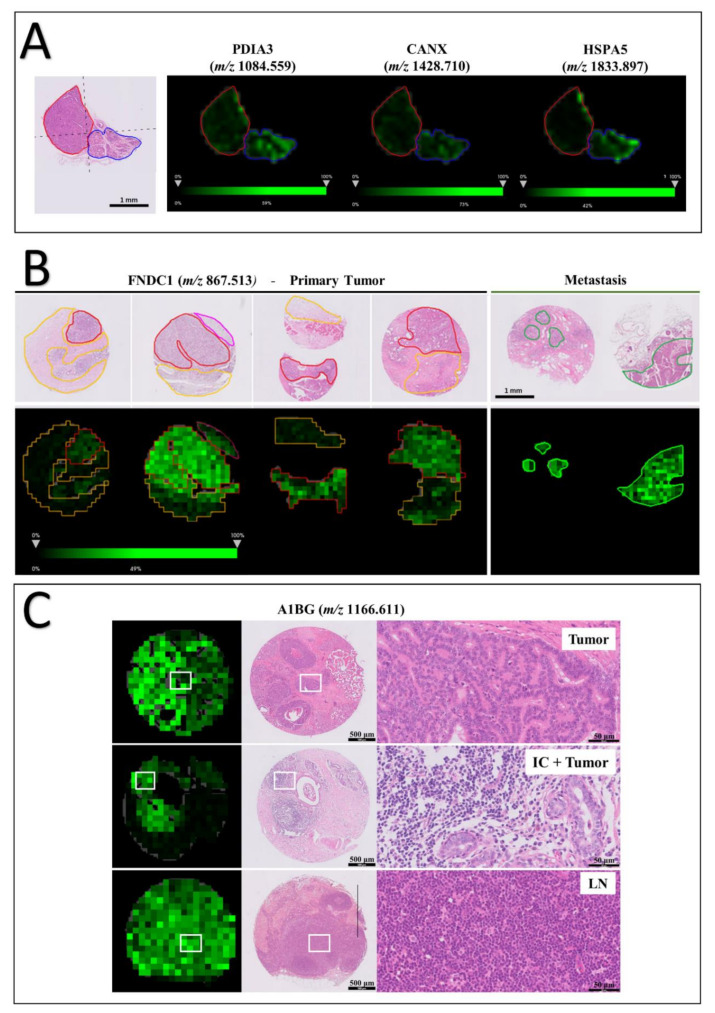
MSI data of canine samples. (**A**) WD (blue line) and PD (red line) tissue distribution of *m/z* signals assigned to proteins enriched in protein folding in endoplasmic reticulum, and folding, assembly, and loading of MHC class I pathway: protein disulfide isomerase family A member 3 (*m/z* 1084.559), calnexin (*m/z* 1215.622) and heat-shock protein family A member 5 (*m/z* 2042.051). (**B**) Signal of FNDC1 peptide at *m/z* 867.513 is strong across primary tumor and metastatic areas. (**C**) A1BG peptide at *m/z* 1166.611 was found to be expressed not only across primary tumor areas but also by inflammatory cells (IC) in canine samples. Corresponding HE staining shows signal distribution in tumor cells, tumor areas with infiltrating inflammatory cells, and across the lymph node (LN).

A1BG, which absolute intensity was higher in the malignant phenotype, was significant for three different pathways involved with platelet mechanisms, such as platelet activation, aggregation, and degranulation. In addition, calnexin (CANX), heat-shock protein family A member 5 (HSPA5), and protein disulfide isomerase family A member 3 (PDIA3), which in GO enriched for protein processing in endoplasmic reticulum, also played an important role for antigen presentation associated to the class I MHC, presenting the second-highest fold enrichment value among all significant pathway classes (Table 2 and Figure 2B). All three proteins were found to be less intense in PD tumor regions (Figure 3A).

### 3.4. Tissue Distribution of FNDC1 and A1BG Peptides

Next, we further explored the spatial distribution of m/z signals with a higher intensity in PD regions and whose identities could be assigned from the LC-MS/MS data. First, we compared ion intensities between tumor regions presenting an aggressive morphological phenotype with non-neoplastic mammary gland tissues. Both peptides at *m/z* 867.513 and 1166.611, respectively assigned to FNDC1 and A1BG proteins, were significantly more intense in PD tumor over control regions (*p* = 0.0006 for *m/z* 867.513 and *p* = 0.0395 for *m/z* 1166.611). Then, we examined tissue distributions of both peaks across the entire sample, including tumor microenvironment, lymph nodes, and metastatic sites. Besides tumor regions, the FNDC1 peptide was also of high intensity across metastatic areas in lymph nodes and other secondary tumor sites, as lungs and abdominal masses (Figure 3B). Similarly, the A1BG peptide was detected across primary tumor tissue in both WD and PD regions, as well as in immune cell clusters, as demonstrated in Figure 3C. Ion abundance varied between intratumor infiltrating cells and tissue-resident leukocytes in the lymph nodes. In the latter, the peptide distributions seemed anatomically homogeneous, since no distinction between cortex and medulla could be observed.

### 3.5. Prognostic Value of FNDC1, A1BG, CANX, HSPA5 and PDIA3 in Human Breast Cancer Patients

**Figure 4:**
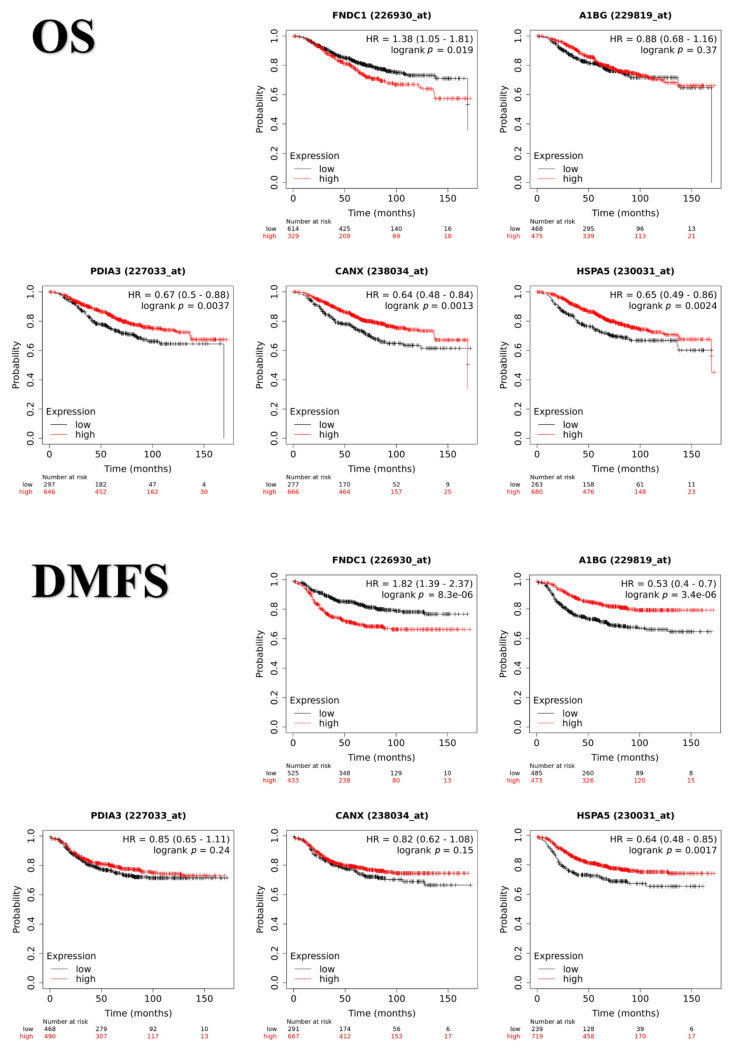
Prognostic values of FNDC1, A1BG, PDIA3, CANX and HSPA5 for OS and DMFS in human breast cancer patients. Gene expression and clinical outcome data were analyzed using Kaplan–Meier plotter. Patients with expression above the threshold are indicated in red line, and patients with expression below the threshold in black line. HR = hazard ratio.

As demonstrated by molecular tissue distributions and functional enrichment analyses, FNDC1, A1BG, CANX, HSPA5, and PDIA3 were related to the immune system response against tumor cells and/or cancer spreading to distant sites in canine cancer, and thus being promising candidates for further validation as comparative prognostic biomarkers. Therefore, we intended to study the relationship between gene expression of such proteins and clinical outcomes of human breast cancer patients using the Kaplan–Meier plotter online database. In silico validation explored the overall survival (OS, *n* = 943 patients) and distant metastasis-free survival (DMFS, *n* = 958 patients) prognostic values that were obtained according to the low and high expression of each gene. We observed that the high expression of FNDC1 (probe 226930_at, *p* = 8.3 × 10^−^^6^), as well as the low expression of A1BG (probe 229819_at, *p* = 3.4 × 10^−^^6^) and HSPA5 (probe 230031_at, *p* = 0.0017) were associated with worse DMFS. In addition, high expression of FNDC1 (probe 226930_at, *p* = 0.019) and the low expression of PDIA3 (probe 227033_at, *p* = 0.0037), HSPA5 (probe 230031_at, *p* = 0.0024) and CANX (probe 238034_at, *p* = 0.0013) were also associated with a worse overall survival in breast cancer patients (Figure 4).

## 4. Discussion

In the present study, we performed MSI to assess and compare peptide expression in 282 cancer regions annotated within 17 tumor samples from five different patients, minimizing the effect of genetic background and macroenvironment influence. By analyzing heterogeneous intratumor subpopulations and metastatic sites, we demonstrated that FNDC1, A1BG, CANX, HSPA5, and PDIA3 may be key factors to tumor malignancy in a canine model of breast cancer. A strong indication would be that peptides assigned to these proteins were not only significant to discriminate between phenotypes, but they also presented a significant prognostic value, as demonstrated in the survival analysis based on gene expression data of human breast cancer.

The degree of differentiation generates relevant information regarding clinical behavior, and it still has a direct impact to determine patient diagnosis and prognosis [22,23,24,25]. Here, the histological grading system was used to divide intratumor populations into two distinct groups based on their level of differentiation: well- and poorly differentiated tumor regions (WD and PD). A total of 168 *m/z* signals were able to discriminate between WD and PD groups, in which the majority of ions presented a lower intensity in tumor regions with a more malignant morphological phenotype. As it may be, a small part of this effect might have been due to a greater amount of stroma elements in WD populations, since well-differentiated breast carcinomas do not show as many solid grow patterns as in PD regions. However, the importance of such molecules in the context of tumor development and progression should not be diminished. It is well known that the tumor microenvironment, which includes extracellular matrix (ECM) molecules, inflammatory cells, cancer-associated fibroblast, and blood vessels, is closely associated with carcinogenesis, cell proliferation, survival, invasion, and metastasis [8,26,27]. Collagen, for example, is one of the most abundant ECM proteins and withstands consecutive stages of degradation, redeposition, and remodeling, where both the increase and decrease in its deposition may be associated with the increase in tumor malignancy [28,29,30]. Here, peptides assigned to collagens types I, VI, and XII were recurrent in several significant terms in GO and pathway enrichment analysis, representing an important link between tumor microenvironment and cancer cells.

Peptides with a lower intensity in PD populations were assigned to proteins involved in molecular functions such as the coagulation cascade, ECM structure, and cell adhesion. Interestingly, we also observed a decreased expression in PD regions of *m/z* signals assigned to proteins such as calnexin, HSPA5, and PDIA3, which enriched for protein processing in the endoplasmic reticulum (ER) and peptide folding, assembly, and loading of class I MHC. Cells undergoing malignant progression have increased proliferation and the ability to adapt under adverse environments, frequently leading to ER stress resulting in protein misfolding, reduced ER processing, and the unfolded protein response (UPR) activation [31,32]. In normal physiology, misfolded and mutated proteins are cleaved into peptides which will be transported from the cytosol to the ER and later loaded into the MHC complex for antigen presentation triggering an immune response through CD8+ T cells [33]. Hence, losses or defects in transport and peptide loading in MHC may disrupt and impede antigen processing and presentation, thus having a profound effect on patient therapy and tumor cell survival against the immune system [34]. Accordingly, in our study, the low gene expression of calnexin, HSPA5, and PDIA3 had a significant prognostic value for OS and DMFS in human breast cancer patients.

PDIA3 interacts with lectin chaperones calreticulin and calnexin to modulate folding of newly synthesized glycoproteins [35], and the low expression of PDIA3 has been associated with poor overall survival for non-small lung cancer and gastric cancer, due to the formation of a complex with MHC class I [35,36,37], corroborating with our results. HSPA5 (also known as BiP) is a master regulator of the UPR [38]. Fae and collaborators demonstrated PDIA3 and HSPA5 to be recognized by heart infiltrating and peripheral T cells in chronic rheumatic heart disease patients, suggesting that these proteins may be involved in an autoimmune-mediated tissue response as autoantigen targets of antibodies [39]. In cancer, future studies could be performed to determine whether the same mechanism also participates in tumor progression and metastasis due to evasion from infiltrating immune cells.

Moreover, during protein folding, HSPA5 associates with calnexin, a highly abundant transmembrane chaperone that folds synthesized glycoproteins in the ER lumen, playing a central role in glycoprotein quality control [40,41,42,43,44]. The upregulation of calnexin has been associated with a worse prognosis in several types of cancer, but results are still controversial. Although its increased expression was shown to negatively regulate tumor MHC I surface expression and promote STAT3 oncogene activation leading to tumor malignancy [45,46], calnexin expression levels were significantly higher only in Stage I lung cancer patients when compared to healthy controls, while in colorectal cancer (CRC), no associations were found between higher expressions of calnexin in CRC Stages II or III compared to normal tissue samples [47,48]. Additionally, low or defective expression of calnexin in primary breast cancer was reported to be associated with a higher risk of brain metastases, due to defects in T-cell-based immunosurveillance [49]. These results corroborate our theory that downregulation of PDIA3, HSPA5, and calnexin, at a certain point of tumor progression, may contribute to malignancy by promoting tumor-immune system evasion through a dysregulation in peptide processing and antigen presentation, having a direct impact on patient outcome and therapy efficiency.

Among the six peptides with a higher intensity in PD regions, tissue distribution of *m/z* signal at 867.513, assigned to the Fibronectin type III domain-containing 1 protein (FNDC1, also known as ASG8), revealed that this ion was not only more intense in tumors over control regions, but also highly expressed across metastatic sites. In addition, its high expression was associated with a shorter DMFS in human patients. The fibronectin III domain-containing proteins are found in tandem arrays in ECM proteins such as fibronectin and tenascin [50], sharing an identical protein fold similarly to the immunoglobulin domain, but with a limited amino acid sequence identity [51]. Although fibronectin III domains role in cell adhesion and migration has been described [52,53], specifically FNDC1 expression and function still remain poorly understood. In normal tissues, FNDC1 seems to be involved in angiogenic events, such as hypoxia-induced apoptosis of cardiomyocytes and vascular epidermal growth factor-mediated signal processing during angiogenesis [54,55]. In cancer, FDNC1 expression was shown to be altered by deregulated epigenetic CpG island methylation in salivary gland adenoid cystic carcinomas [56]. In addition, FNDC1 knockdown inhibited gastric and prostate tumor cell proliferation, invasion and also downregulated the expression of important proteins involved in epithelial-to-mesenchymal transition (EMT), but the mechanisms have still not been elucidated [57,58]. These findings, combined with the results observed in our work, strongly suggest that FNDC1 may be a potential cancer biomarker candidate. To our knowledge, this is the first report to suggest that FNDC1 may also play an important role in breast cancer metastatic disease.

Besides FNDC1, LC-MS/MS analysis of peptides with a higher intensity in PD also identified a signal corresponding to the Alpha-1B-glycoprotein (A1BG). Significantly enriching for pathways of the coagulation cascade, tissue distribution of the *m/z* signal at 1166.611 showed by MSI analysis demonstrated that A1BG peptide was mainly expressed by inflammatory cells infiltrating tumor areas and also in the lymph nodes. Additionally, the low expression of A1BG showed to predict a worse DMFS prognosis in human breast cancer patients. A1BG, with still unknown functions, shows homology to the immunoglobulin supergene family and has been implicated in immune response inflammation processes [59,60,61]. Mainly found in the serum, plasma, and other bio-fluids such as pancreatic juice and urine, A1BG expression has been pointed out as a relevant biomarker for several types of cancer [62,63,64,65,66]. A1BG together with Complement C3 was shown to be overexpressed in serum samples from patients with squamous cell carcinoma of the cervix and grade III cervical intraepithelial cancer, compared to healthy control women [67,68]. Moreover, sialylation changes of A1BG and Complement C3 were demonstrated in blood samples of control and breast cancer patients, suggesting that such modification in the protein glycosylation pattern of these proteins may also serve as a potential biomarker [69]. The capacity of tumor cells to produce and release A1BG is still uncertain. To date, only a few studies have demonstrated its expression in cancer cells [64,67], and therefore, further experiments are needed to validate A1BG expression and prognostic significance in tumor tissue samples.

## 5. Conclusions

Through the comparison of the proteomic profile of intratumor cell populations in different degrees of differentiation, we demonstrated that the malignant phenotype may have arisen as a consequence of alterations in the expression of key proteins such as FNDC1, A1BG, CANX, HSPA5, and PDIA3 and that most of these variations may be involved in tumor evasion against inflammatory cells facilitating cancer spreading. A strong indication would be that all five proteins were presented as significant prognostic values for overall survival and metastasis-free survival in human patients, as demonstrated in an independent validation. Now, further investigations are necessary to deeply characterize how such variations evolve with malignancy. Therefore, dog samples are useful proof of concept experiments, and novel comparative studies can be applied for many other types of cancer, and other disease conditions.

## Figures and Tables

**Table 1:** Biological processes and molecular function terms enriched in GO analysis using 38 proteins IDs retrieved from LC/MS-MS data.

Biological Process	Fold Enrichment	IDs	FDR
Protein folding in endoplasmic reticulum	>100	CANX, HSPA5, PDIA3	2.7 × 10^−2^
Extracellular matrix structural constituent conferring tensile strength	52.16	COL1A1, COL1A2, COL6A3, COL12A1	1.73 × 10^−3^
Extracellular matrix structural constituent	18.98	COL1A1, COL1A2, COL6A3, COL12A1, VCAN, EMILIN2	1.3 × 10^−3^
Structural molecule activity	6.81	COL1A1, COL1A2, COL6A3, COL12A1, VCAN, EMILIN2, LMNA, EPB41L2, CTNNA1	4.95 × 10^−3^
Cadherin binding Cell adhesion molecule binding	17.75 11.01	LIMA1, EEF1D, CALD1, TNKS1BP1, HSPA5, FLNA, TAGLN2, SERBP1, DDX3X, CTNNA1, BAG3	1.07 × 10^−7^ 9.50 × 10^−6^

**Table 2:** Significant REACTOME enriched pathways. Results are sorted by the hierarchical relations between over-represented functional classes, which may be interpreted as a group rather than individually.

Pathway Name	Fold Enrichment	IDs	FDR
GP1b-IX-V activation signaling	>100	COL1A1, COL1A2, FLNA	2.47 × 10^−3^
Platelet activation signaling and aggregation	12.39	COL1A1, COL1A2, FLNA, A1BG, HSPA5, TAGLN2	4.13 × 10^−3^
Antigen presentation: folding, assembly and peptide loading of class I MHC	61.69	CANX, HSPA5, PDIA3	4.85 × 10^−3^
Collagen chain trimerization	48.60	COL1A1, COL1A2, COL6A3, COL12A1	4.34 × 10^−3^
Collagen biosynthesis and modifying enzymes	31.92	COL1A1, COL1A2, COL6A3, COL12A1	3.51 × 10^−3^
Collagen formation	24.03	COL1A1, COL1A2, COL6A3, COL12A1	5.58 × 10^−3^
Assembly of collagen fibrils and other multimeric structures	35.64	COL1A1, COL1A2, COL6A3, COL12A1	4.63 × 10^−3^
Collagen degradation	33.42	COL1A1, COL1A2, COL6A3, COL12A1	4.43 × 10^−3^
Degradation of the extracellular matrix	15.28	COL1A1, COL1A2, COL6A3, COL12A1	2.41 × 10^−2^
ECM proteoglycans	28.14	COL1A1, COL1A2, COL6A3, VCAN	4.83 × 10^−3^
Platelet degranulation	16.84	A1BG, HSPA5, FLNA, TAGLN2	1.95 × 10^−2^
Response to elevated platelet cutosolic Ca2+	16.20	A1BG, HSPA5, FLNA, TAGLN2	2.08 × 10^−2^
Signaling by receptor tyrosine kinases	8.19	COL1A1, COL1A2, COL6A3, STMN1, HNRNPM, CSN2, CTNNA1	5.38 × 10^−3^

## Data Availability

The data presented in this study are available on request from the corresponding author.

## Supplementary Materials

The following are available online at https://www.mdpi.com/article/10.3390/cancers13235901/s1. Table S1: Patient Information, Table S2: Discriminative analysis, Table S3: Identity Assignment.
