# Supplementary material for: Proteomic Analysis Identifies FNDC1, A1BG, and Antigen Processing Proteins Associated with Tumor Heterogeneity and Malignancy in a Canine Model of Breast Cancer
Source: Cancers (Basel). 2021 Nov 24;13(23):5901. doi: 10.3390/cancers13235901 (PMC8657005; doi:10.3390/cancers13235901)
Supplement: Supplementary file 1 [file cancers-13-05901-s001.zip › cancers-1439835-supplementary.pdf]

Supplementary Table S1 - Patient Information

Patient	Age (years)	Breed	Status	Sample	Tumor	Histopathological classification
PA1249	9	Labrador Retriever	Unknown cause of death	58/17	1	Anaplastic carcinoma
					-	Carcinoma e malignant myoepithelioma**
					-	Anaplastic carcinoma**
3255/ 8448	12	Daschund	Death due to tumor	61/17	2	Complex carcinoma
					3	Complex carcinoma
					4	Mixed-type carcinoma
					-	Simple adenoma**
					5	Complex carcinoma
					6	Complex carcinoma
					7	Mixed-type carcinoma
					8	Mixed-type carcinoma
					9	Complex carcinoma
3528/ 8432	12	Poodle	Alive*	63/17	10	Mixed-type carcinoma
					11	Simple carcinoma
					12	Mixed-type carcinoma
					13	Simple carcinoma tubular type
					14	Benign mixed-type
066/18	12	Brazilian Mastiff	Death due to tumor	66/18	15	Invasive Micropapillar carcinoma
					16	Invasive Micropapillar carcinoma
PA1796	13	Mixed Breed	Death due to tumor	71/18	17	Simple carcinoma tubular type

* Until last contact with the owner > 400 days after surgery.

** Tumor not included in the study due to tissue processing issues

Supplementary Table S2 - Discriminative analysis

m/z	AUC	FDR	Fold Change	log2 FC
1863.858	0.76	4.31E-15	-1.782408154	-0.83383
2104.043	0.76	2.23E-12	-1.752899149	-0.80974
2216.036	0.76	5.14E-15	-1.862037864	-0.89688
1585.763	0.75	2.76E-14	-2.037509717	-1.02681
2056.961	0.75	1.19E-12	-2.097088463	-1.06839
2131.094	0.75	2.29E-13	-1.908148197	-0.93217
1095.561	0.74	1.29E-13	-2.11731433	-1.08224
1508.677	0.74	1.45E-15	-1.797401081	-0.84591
1576.799	0.74	2.13E-12	-1.830989872	-0.87262
1586.736	0.74	3.29E-11	-2.329251963	-1.21987
1607.744	0.74	1.30E-12	-1.871561054	-0.90424
1655.785	0.74	2.62E-12	-1.894616544	-0.92191
2120.034	0.74	1.61E-13	-2.156605513	-1.10876
2153.079	0.74	2.09E-13	-2.160092346	-1.11109
2853.371	0.74	4.31E-15	-1.707898704	-0.77222
2856.382	0.74	8.53E-15	-2.037731265	-1.02696
1096.565	0.73	2.00E-13	-2.088640405	-1.06256
1289.652	0.73	4.45E-11	-1.631715792	-0.70639
1332.649	0.73	1.73E-13	-2.212737563	-1.14583
1407.654	0.73	1.07E-13	-1.775670132	-0.82836
1453.736	0.73	1.36E-11	-2.417976116	-1.2738
1473.663	0.73	2.12E-12	-1.79230365	-0.84182
1560.805	0.73	2.42E-11	-2.552094446	-1.35168
1706.763	0.73	1.62E-11	-2.021276191	-1.01527
1735.826	0.73	1.04E-12	-1.912496083	-0.93546
1812.871	0.73	1.63E-12	-1.881096413	-0.91157
1818.891	0.73	2.27E-12	-2.040937323	-1.02923
1832.848	0.73	6.08E-12	-2.143523185	-1.09998
1834.889	0.73	3.56E-11	-2.038655602	-1.02762
1836.859	0.73	1.48E-11	-2.338891145	-1.22582
1839.872	0.73	2.10E-12	-1.753104247	-0.80991
1908.903	0.73	1.29E-13	-1.975079385	-0.98191
2252.1	0.73	4.31E-15	-1.710749993	-0.77463
2342.172	0.73	4.92E-13	-1.791873818	-0.84147
2343.165	0.73	1.74E-12	-1.988564714	-0.99173
2705.225	0.73	2.70E-13	-1.540011171	-0.62294
2706.231	0.73	9.04E-13	-1.994603039	-0.9961
3188.484	0.73	7.26E-14	-2.2346746	-1.16006
710.4064	0.72	1.86E-12	-2.165326892	-1.11458
710.7411	0.72	5.95E-13	-2.19855789	-1.13656
1054.499	0.72	5.73E-14	-2.155516669	-1.10803
1111.593	0.72	2.42E-12	-1.872440246	-0.90492
1115.541	0.72	4.19E-15	-1.93199861	-0.95009
1257.616	0.72	3.64E-13	-1.89692901	-0.92367
1267.67	0.72	3.73E-11	-1.957436959	-0.96897
1279.6	0.72	2.42E-12	-1.831583888	-0.87309
1428.711	0.72	4.68E-13	-1.770337087	-0.82402

1465.684	0.72	5.26E-11	-1.852156443	-0.88921
1558.685	0.72	1.91E-10	-1.695274127	-0.76152
1566.741	0.72	6.21E-11	-2.214216349	-1.1468
1636.83	0.72	3.70E-15	-1.777303256	-0.82969
1653.833	0.72	3.27E-12	-2.087167826	-1.06155
1677.767	0.72	8.99E-12	-2.127095802	-1.08889
1727.767	0.72	2.08E-14	-2.157350297	-1.10926
1728.771	0.72	3.70E-15	-1.796585963	-0.84526
1758.725	0.72	5.06E-12	-2.739438091	-1.45388
1786.771	0.72	1.94E-13	-2.10293502	-1.0724
1798.805	0.72	2.00E-11	-2.162163782	-1.11248
1819.886	0.72	4.64E-13	-2.375613544	-1.2483
1885.84	0.72	1.94E-12	-2.329867141	-1.22025
1953.887	0.72	5.60E-15	-1.77172587	-0.82516
2019.96	0.72	4.01E-15	-2.514971454	-1.33054
2114.076	0.72	8.53E-12	-2.436446528	-1.28478
2423.143	0.72	8.58E-12	-1.665609768	-0.73605
2822.328	0.72	3.65E-12	-2.016418859	-1.0118
842.4093	0.71	1.34E-13	-2.180662111	-1.12477
868.4613	0.71	1.54E-11	-2.034757308	-1.02486
970.4753	0.71	1.24E-11	-1.982509049	-0.98733
1081.583	0.71	7.67E-13	-1.718701856	-0.78132
1132.482	0.71	1.88E-10	-2.103564027	-1.07284
1258.621	0.71	3.33E-12	-1.731107256	-0.7917
1264.535	0.71	7.22E-11	-1.703586433	-0.76858
1289.582	0.71	1.19E-12	-1.835367143	-0.87607
1401.639	0.71	3.13E-13	-2.040098371	-1.02864
1429.635	0.71	2.94E-12	-2.132185941	-1.09233
1475.716	0.71	1.44E-12	-1.927863939	-0.947
1530.659	0.71	1.86E-12	-2.760398079	-1.46488
1563.821	0.71	7.94E-12	-2.311996741	-1.20914
1588.725	0.71	1.17E-11	-1.935277548	-0.95254
1593.752	0.71	5.72E-11	-2.02521381	-1.01807
1598.782	0.71	1.50E-10	-2.035992299	-1.02573
1684.828	0.71	8.36E-16	-1.904133039	-0.92913
1694.86	0.71	1.29E-14	-2.533910746	-1.34137
1720.873	0.71	4.20E-11	-1.957112765	-0.96873
1827.893	0.71	1.07E-12	-1.908857265	-0.93271
1848.841	0.71	3.04E-10	-2.410070181	-1.26908
1992.92	0.71	6.61E-12	-2.099158108	-1.06981
2002.989	0.71	3.08E-11	-1.75337283	-0.81013
2078.946	0.71	1.07E-11	-1.58967033	-0.66873
2081.946	0.71	1.26E-12	-2.185521303	-1.12798
2089.017	0.71	6.03E-11	-2.250529674	-1.17026
2169.952	0.71	5.60E-15	-2.163148589	-1.11313
2195.977	0.71	8.73E-12	-2.094415936	-1.06655
2214.946	0.71	6.85E-10	-1.876982895	-0.90842
2219.961	0.71	3.21E-13	-1.60199639	-0.67987
2242.965	0.71	7.31E-15	-1.474767921	-0.56049
2316.029	0.71	4.74E-12	-2.033432147	-1.02392

2719.21	0.71	8.73E-12	-2.274932047	-1.18582
679.5474	0.70	2.27E-11	1.794552452	0.843624
685.6922	0.70	2.58E-11	-2.220920385	-1.15116
686.0273	0.70	5.64E-12	-2.146313873	-1.10186
742.3051	0.70	1.31E-10	1.787413704	0.837874
758.3778	0.70	2.92E-12	-1.772951415	-0.82615
781.3937	0.70	1.49E-11	-1.894860591	-0.92209
785.3886	0.70	1.48E-11	-1.979111834	-0.98485
836.4349	0.70	4.60E-11	-2.091066236	-1.06424
840.4664	0.70	1.76E-10	-1.667996007	-0.73812
867.513	0.70	3.84E-11	1.695851605	0.76201
886.4352	0.70	9.47E-10	-1.939518292	-0.9557
898.5077	0.70	6.38E-10	-1.825972946	-0.86867
1041.613	0.70	1.63E-10	2.092501402	1.065229
1084.56	0.70	1.14E-11	-2.058165556	-1.04136
1088.534	0.70	1.33E-09	-1.978800583	-0.98463
1096.516	0.70	8.90E-11	-1.986334319	-0.99011
1117.543	0.70	8.35E-11	-1.718707925	-0.78132
1120.606	0.70	3.29E-11	-1.712481694	-0.77609
1128.557	0.70	2.26E-10	-1.678128526	-0.74685
1131.531	0.70	2.03E-11	-1.633727273	-0.70817
1142.589	0.70	1.32E-11	-1.59694879	-0.67532
1158.647	0.70	4.05E-11	2.427134903	1.279254
1165.555	0.70	1.29E-11	-1.855902849	-0.89212
1166.612	0.70	2.90E-12	-1.926180295	-0.94574
1177.564	0.70	1.21E-12	-2.114972411	-1.08064
1184.489	0.70	1.77E-10	-1.886457679	-0.91568
1201.58	0.70	1.96E-11	-2.056285389	-1.04004
1214.587	0.70	2.14E-12	-1.796711563	-0.84536
1226.643	0.70	2.69E-11	-2.021024943	-1.01509
1258.621	0.70	1.25E-10	-2.28877864	-1.19458
1262.627	0.70	2.47E-09	-2.052750298	-1.03756
1311.563	0.70	9.54E-12	-2.428696776	-1.28018
1337.674	0.70	2.42E-11	-1.798589667	-0.84687
1440.7	0.70	3.64E-16	-2.089603072	-1.06323
1487.664	0.70	1.87E-09	-2.632601772	-1.39649
1496.649	0.70	6.55E-11	-2.171717938	-1.11884
1538.711	0.70	1.36E-11	-2.454496626	-1.29543
1642.732	0.70	3.89E-12	-1.881915779	-0.9122
1649.773	0.70	3.58E-08	-2.644381216	-1.40293
1678.773	0.70	1.54E-11	-2.238867161	-1.16277
1700.737	0.70	7.05E-12	-2.020732664	-1.01488
1728.74	0.70	1.31E-08	-2.548759245	-1.3498
1744.759	0.70	2.01E-11	-1.793015604	-0.84239
1749.848	0.70	1.20E-10	-1.849200089	-0.8869
1750.852	0.70	2.07E-10	-2.051696567	-1.03682
1756.762	0.70	3.64E-10	-2.452817062	-1.29444
1757.804	0.70	8.09E-12	-2.201993109	-1.13881
1833.897	0.70	5.16E-11	-2.027074326	-1.0194
1856.845	0.70	4.55E-10	-2.12656151	-1.08852

1888.903	0.70	3.65E-12	-2.070260071	-1.04981
2041.015	0.70	2.61E-09	-1.83556151	-0.87622
2063.928	0.70	2.28E-12	-2.033521306	-1.02398
2110.006	0.70	5.49E-09	-2.345987049	-1.2302
2198.948	0.70	8.73E-12	-3.350626646	-1.74443
2257.059	0.70	6.42E-12	-1.729437342	-0.7903
2268.091	0.70	3.56E-11	-1.49965247	-0.58463
2272.031	0.70	3.80E-11	-1.602684931	-0.68049
2274.077	0.70	2.13E-12	-1.561091551	-0.64256
2280.042	0.70	1.25E-10	-1.99419825	-0.99581
2290.07	0.70	1.22E-10	-1.792715957	-0.84215
2301.035	0.70	3.10E-09	-1.981822325	-0.98683
2344.15	0.70	1.55E-11	-1.793312689	-0.84263
2420.153	0.70	9.14E-11	-1.677025554	-0.7459
2435.176	0.70	1.42E-11	-1.808032239	-0.85442
2437.16	0.70	4.95E-09	-2.280424736	-1.1893
2549.193	0.70	2.98E-12	-1.855817041	-0.89205
2611.221	0.70	2.51E-09	-2.220252084	-1.15072
2703.216	0.70	6.50E-13	-2.153325003	-1.10657
2820.339	0.70	7.84E-11	-2.157007346	-1.10903
2876.364	0.70	1.34E-13	1.974112187	0.981204

Supplementary Table S3 - Identity assignment

	Mass matching - LC-MS (DE m/z)			
**Observed m/z (MSI)**	**Theoretical m/z (LCMS)**	**Error (ppm)**	**Protein**	**Symbol**
836.4349428	836.43733	2.853980804	Collagen alpha-1(I) chain	COL1A1
840.4664466	840.46863	2.597885254	Collagen alpha-2(I) chain	COL1A2
867.513017	867.50468	-9.61036458	Fibronectin type III domain containing 1	FNDC1
868.4613165	868.46354	2.560234594	Collagen alpha-2(I) chain	COL1A2
886.4351666	886.43772	2.880472211	Collagen alpha-1(I) chain	COL1A1
1041.612806	1041.60512	-7.378986012	Keratin 19	KRT19
			IF rod domain-containing protein	KRT18
1084.559882	1084.56734	6.876901254	Protein disulfide-isomerase	PDIA3
1096.516204	1096.50917	-6.414695994	Peptidylprolyl isomerase	FKBP10
1111.592518	1111.59937	6.164449562	SERPINE1 mRNA binding protein 1	SERBP1
1120.606475	1120.61093	3.97595533	T-complex protein 1 subunit gamma	CCT3
1128.557115	1128.56438	6.437154662	Erythrocyte membrane protein band 4.1 like 2	EPB41L2
1131.531114	1131.52766	-3.052711208	Lamin A/C	LMNA
1165.555324	1165.54839	-5.949528676	Stathmin	STMN1
1166.611827	1166.6092	-2.251610591	Alpha-1-B glycoprotein	A1BG
1177.563935	1177.55242	-9.779009966	Versican	VCAN
1184.488719	1184.49535	5.597887581	Caldesmon 1	CALD1
1201.579621	1201.58815	7.098249529	Insulin like growth factor binding protein 5	IGFBP5
1214.586772	1214.576	-8.869017204	LIM domain and actin binding 1	LIMA1
1226.643484	1226.6528	7.594906684	Filamin A	FLNA
1258.620978	1258.62737	5.078729227	Tankyrase 1 binding protein 1	TNKS1BP1
1279.599711	1279.59132	-6.557441379	Transgelin	TAGLN2
1289.581578	1289.5869	4.126590252	DEAD-box helicase 3 X-linked	DDX3X
1311.563208	1311.56992	5.117584025	HATPase_c domain-containing protein	HSP90AB1
			Heat shock protein 90 alpha family class A member 1	HSP90AA1
1332.648959	1332.639	-7.473516054	Tropomyosin 1	TPM1
			Tropomyosin 2	TPM2
1337.674103	1337.67696	2.136004974	Purine nucleoside phosphorylase	PNP
1401.639318	1401.64923	7.07185018	Elastin microfibril interfacer 2	EMILIN2
1428.710805	1428.70775	-2.138079695	Calnexin	CANX
1430.638557	1430.64341	3.392254692	peptidyl-prolyl cis-trans isomerase NIMA-interacting 4	PIN4
1473.663429	1473.65644	-4.742353625	BCL2 associated athanogene 3	BAG3
1508.676561	1508.68634	6.481823094	A-kinase anchoring protein 12	AKAP12
1558.684573	1558.69547	6.991339514	Eukaryotic translation elongation factor 1 delta	EEF1D
1560.804534	1560.81288	5.347468615	Collagen alpha-1(I) chain	COL1A1
1585.762918	1585.77174	5.563455753	Beta-casein	CSN2
1598.781714	1598.79081	5.689435818	Marginal zone B and B1 cell specific protein	MZB1
1636.829666	1636.83044	0.472705291	Tubulin beta chain	TUBB4B
			Tubulin beta chain	TUBB
			Tubulin beta chain	TUBB2A
			Tubulin beta 1 class VI	TUBB1
1653.833087	1653.84424	6.743970629	Collagen type XII alpha 1 chain	COL12A1
1812.871458	1812.86101	-5.763458468	ADP ribosylation factor GTPase activating protein 3	ARFGAP3
1818.891464	1818.88281	-4.758039953	Catenin alpha 1	CTNNA1
1833.897314	1833.90897	6.355870521	Heat shock protein family A (Hsp70) member 5	HSPA5
1839.872016	1839.88401	6.518749375	Heterogeneous nuclear ribonucleoprotein M	HNRNPM
1888.903191	1888.91094	4.102141475	Jupiter microtubule associated homolog 2	JPT2
1908.902626	1908.91584	6.922255907	Uncharacterized protein	NA
2002.988775	2003.00533	8.26514225	Collagen alpha-1(I) chain	COL1A1
2056.961085	2056.96443	1.626021729	Collagen type XII alpha 1 chain	COL12A1
2063.928283	2063.93049	1.069208615	Transglutaminase 2	TGM2
2089.016734	2089.00927	-3.573120401	Collagen type VI alpha 3 chain	COL6A3
2104.043339	2104.05569	5.86999721	PDZ domain-containing protein	AHNAK2
2110.006386	2110.00472	-0.789684191	Collagen type XII alpha 1 chain	COL12A1
2153.079398	2153.0808	0.651090944	Collagen type XII alpha 1 chain	COL12A1
2344.150356	2344.14108	-3.956972897	Lipocln_cytosolic_FA-bd_dom domain-containing protein	OBP2B
3188.483712	3188.48256	-0.361273044	Caldesmon 1	CALD1
